# Commentary: AI-based preeclampsia detection and prediction with electrocardiogram data

**DOI:** 10.3389/fcvm.2024.1437369

**Published:** 2024-07-30

**Authors:** Lionel Carbillon

**Affiliations:** ^1^ Department of Obstetrics and Gynecology, Hôpital Jean Verdier, Assistance Publique—Hôpitaux de Paris, Bondy, France; ^2^ Sorbonne North Paris University, Bobigny, France

**Keywords:** preeclampsia, gestational cardiovascular adaptation, blood pressure, cardiac output, artificial-intelligence, electrocardiogram, fetal growth restriction

A Commentary on AI-based preeclampsia detection and prediction with electrocardiogram data By Butler L, Gunturkun F, Chinthala L, Karabayir I, Tootooni MS, Bakir-Batu B, Celik T, Akbilgic O, Davis RL. (2024). Front. Cardiovasc. Med. 11:1360238. doi: 10.3389/fcvm.2024.1360238

## 1 Introduction

Preeclampsia is the final systemic manifestation of poor adaptation of the maternal body to pregnancy (1, 2).

Early gestational adaptation of maternal organism is a consequence and reflection of the first few weeks of pregnancy and quality of placentation. In particular, early adjustments in vascular tone, volume expansion, cardiac output, and blood pressure are crucial, and reflect adaptations of the utero-placental circulation and cardiovascular system caused by the hemochorial nature of placentation in our species (3–5).

In all clinical forms of preeclampsia, the exceeding of the capacities of the circulatory system occurs “at a given moment” in the progression of the underlying pathophysiological process leading towards cardiac output decrease (6, 7), vasoconstrictive tone, hypertension and failure of the maternal organism to the demands of pregnancy, finally leading to preeclamptic syndrome (8, 9). Butler et al. (10) have published an interesting study evaluating a proposed artificial intelligence (AI)-based algorithm for preeclampsia prediction. However, the “moment” (the precise gestational age), at which the clinical emergence of the disease (and even more, severe features of preeclampsia and placenta-related complications) will occur, is extremely difficult to predict with precision, especially without taking into account utero-placental circulation indices. Thus, I believe that the message that “preeclampsia can be identified with high accuracy via application of AI models to electrocardiographic (ECG) data” (10), as claimed by the authors in the conclusion of the abstract, should not be professed. The appropriate message should instead be that “ECG data can help identify pregnant women at high risk of preeclampsia”, as rightly pointed out by the authors at the beginning of the discussion section.

Indeed, defective placentation is the well-recognized primum movens of the underlying pathological process (early “placental” stages of the underlying, pre-clinical process) (1), which can lead to the clinical emergence of preeclampsia when the adaptability of the maternal circulatory system is exceeded (1, 6–8). This also reflects exceeding the immunologic tolerance of the conceptus, after the underlying process of the disease enters its last stages towards a systemic inflammatory response (1, 11, 12), with impaired synthesis of vasorelaxing agents and endogenous anticoagulants, increased production of vasoconstrictors, and the ultimate endothelial dysfunction (13) and its major systemic manifestations. These last stages are associated with inadequate “placental derived factors” in the mother's blood, preceding and promoting the appearance of the complete clinical syndrome. Moreover, the syndrome may occur rapidly or progressively, and also depends on the severity of placental dysfunction, which is not considered by the algorithm.

## 2 Discussion

Thus, the “10-s 12-lead ECG” data (10), which finely assess gestational adaptation of the heart, provides a very useful tool for assessing the possible maladaptation of the maternal cardiovascular system to pregnancy at a given time. However, potential future users of this device should bear in mind that the potential for progression of the underlying disease at a given moment depends not only on the adaptation of the cardiovascular system, but also on placental function (the very origin of the pathology), fetal growth, and placental derived (pro and anti angiogenic) factors in the mother's blood (14, 15).

Notably, this AI-based model, like others (16), has only a modest predictive performance in women with preeclampsia giving birth at less than 37 weeks of gestation [95% area under the curve confidence interval of 0.76 (0.58–0.95)]. This performance should make potential users very cautious in using its supposed negative predictive value, especially since at these gestational ages (especially before 32–34 weeks of gestation), timely preventive treatment for prematurity complications with betamethasone and magnesium sulfate treatments may be necessary and are time-consuming. Therefore, any delay in diagnosis of preeclampsia can lead to a loss of chance for the newborn, if the diagnosis of preeclampsia is made too late to implement preventive treatments of prematurity complications without endangering the mother.

Moreover, fetal growth-restricted pregnancies are characterized by a lower cardiac output and higher total vascular resistance index than that expected for gestation (17, 18). In these circumstances, in accordance with the “Guytonean model”, such “hyperdynamic state makes it possible for extremely slight long-term changes in blood fluid volume and cardiac output to raise or lower arterial pressure” (19–21), which can have serious consequences for the mother and fetus.

As Easterling recently recalled (20), “when cardiac output is under the mean for gestational age and/or vascular resistance is elevated, the association with the development of fetal growth restriction is strong”. Without considering fetal growth restriction (FGR) and possible placental dysfunction, which needs a true targeted expertise of the utero-placental circulation and function (14, 15), the danger of the test lies in its overestimation of the negative predictive value. Such overestimation will inherently impact the relevance of the proposed schedule for follow-up of the patient in the last weeks or months of pregnancy, due to the risk of rapid progression of placental dysfunction and the underlying disease if stages 5–6 have been reached (1) with the possibility of rapid onset of serious complications.

Finally, the combination of various pre-eclampsia risk factors known to “exponentially increase” the risk (22) must also be considered, including the history of early or intermediate preeclampsia, preterm preeclampsia, obesity alone or in combination with chronic hypertension and/or ongoing pregnancy with FGR.
